# The Filum disease and the Neuro-Cranio-vertebral syndrome: definition, clinical picture and imaging features

**DOI:** 10.1186/s12883-020-01743-y

**Published:** 2020-05-11

**Authors:** Miguel B. Royo-Salvador, Marco V. Fiallos-Rivera, Horia C. Salca, Gabriel Ollé-Fortuny

**Affiliations:** 1 Institut Chiari & Siringomielia & Escoliosis de Barcelona, Passeig Manuel Girona 16, 08034 Barcelona, Spain; 2 Anesthesia Department, CIMA Hospital, Barcelona, Spain

**Keywords:** Arnold-Chiari syndrome, Syringomyelia, Scoliosis, Filum terminale

## Abstract

**Background:**

We propose two new concepts, the Filum Disease (FD) and the Neuro-cranio-vertebral syndrome (NCVS), that group together conditions thus far considered idiopathic, such as Arnold-Chiari Syndrome Type I (ACSI), Idiopathic Syringomyelia (ISM), Idiopathic Scoliosis (IS), Basilar Impression (BI), Platybasia (PTB) Retroflexed Odontoid (RO) and Brainstem Kinking (BSK).

**Method:**

We describe the symptomatology, the clinical course and the neurological signs of the new nosological entities as well as the changes visible on imaging studies in a series of 373 patients.

**Results:**

Our series included 72% women with a mean age of 33.66 years; 48% of the patients had an interval from onset to diagnosis longer than 10 years and 64% had a progressive clinical course. The commonest symptoms were: headache 84%, lumbosacral pain 72%, cervical pain 72%, balance alteration 72% and paresthesias 70%. The commonest neurological signs were: altered deep tendon reflexes in upper extremities 86%, altered deep tendon reflexes in lower extremities 82%, altered plantar reflexes 73%, decreased grip strength 70%, altered sensibility to temperature 69%, altered abdominal reflexes 68%, positive Mingazzini’s test 66%, altered sensibility to touch 65% and deviation of the uvula and/or tongue 64%. The imaging features most often seen were: altered position of cerebellar tonsils 93%, low-lying *Conus medullaris* below the T12L1 disc 88%, idiopathic scoliosis 76%, multiple disc disease 72% and syringomyelic cavities 52%.

**Conclusions:**

This is a paradigm shift that opens up new paths for research and broadens the range of therapeutics available to these patients.

## Background

This paper summarizes and culminates the endeavors of various researchers who have been pursuing so far three convergent lines of research: the tethered cord syndrome; the etiopathogenic relationship between Arnold-Chiari Syndrome Type I, Idiopathic Syringomyelia and Idiopathic Scoliosis and other associated pathologies; and finally, the role of spinal cord tethering in the development of Idiopathic Scoliosis.

Even though the first surgical cases of tethered cord release were published already as early as 1857 by Johnson [1] and in 1891 by Jones WL [2], the relationship between tethering of the spinal cord and a certain neurological and spinal symptomatology, i.e. the first concept of tethered cord, was suggested by Fuchs [3] in 1909 in patients with myelomeningocele, as did Lichtenstein [4] later in 1940.

In 1953, Garceau [5] defined the “filum terminale syndrome”, or “cord-traction syndrome”, reporting on three cases with a similar picture, that improved after sectioning of a thick and tight filum terminale, whereas Jones and Love [6] proposed in 1956 the term “tight filum terminale”.

In 1976, Hoffmann [7] was using the term “tethered spinal cord” to define a similar clinical picture, associated to certain radiological criteria such as a low-lying medullary conus and a thick filum terminale.

As a result of these consecutive contributions, after many decades of indecision and difficulties, the tethered cord syndrome was finally described, as caused by an abnormal tethering of the spinal cord by a malformation that is evident on physical examination in the form of a spina bifida, while manifesting by a clinical picture of neurological sensorimotor impairments, predominantly in the lower limbs, associated with frequent deformities of the feet, cutaneous stigmata and genitourinary alterations, according to Fuchs, Lichtenstein and Yamada [3, 4, 8]. The prevalence of a symptomatic tethered cord associated with *spina bifida occulta* was 0.1% of 5499 primary school children in Turkey [9], while generally, all types of spina bifida occur in the range of 0.5–10 per 1000 live births worldwide [10]. The surgical treatment, indicated in 10–20% of cases, consists in the release of the spinal cord that is tethered by the myelo-meningo-vertebral malformation, via a lumbar laminectomy.

On the other hand, in a quite distinct direction of research, many authors observed an association amongst Arnold-Chiari Syndrome Type I, Idiopathic Syringomyelia and Idiopathic Scoliosis over the past decades [11–18] but no pathogenic explanation or possible causal relationship has been sufficiently accepted in order to warrant the initiation of more in-depth studies into this matter.

Finally, a third line of research, very close to our own vision, attempts to explain the pathogenesis of Idiopathic Scoliosis, Arnold-Chiari Syndrome Type I and Basilar Impression with a growth asynchrony between the spine and the spinal cord - a mechanism proposed by Roth in 1981 and 1986 [19, 20]. This would be causing a tethering of the spinal cord, as Porter advanced in 2001 [21, 22], and an excessive and deforming growth of the anterior elements of the thoracic spine, leading to the production of scoliosis with rotation, according to a mechanism suggested by Dickson in 1984 [23]. Some recent magnetic resonance studies, particularly those carried out by Winnie Chou’s team, have identified features which support these theories in IS patients [24], while others, as Milhorat in 2009, have applied these criteria for treatment [25].

Based on the arguments outlined in the doctoral thesis “Contribution to the etiology of syringomyelia” [26], the traction of the spinal cord and brain is proposed as the main mechanism involved in the etiopathogenesis of ISM, ACSI, IS and other diseases also considered idiopathic, such as Platybasia, Basilar Impression, Retroflexed Odontoid and Brainstem Kinking [27, 28].

The objective of this article is to introduce the concept *Neuro-Cranio-Vertebral Syndrome (NCVS)* to define the set of clinical and imaging manifestations that affect the nervous system, the skull and the spine in the form of known diseases like Arnold-Chiari Syndrome Type I, Idiopathic Syringomyelia, Idiopathic Scoliosis and other anomalies like Platybasia, Basilar Impression, Retroflexed Odontoid and Brainstem Kinking. The *Filum Disease* is the most frequent congenital form of the Neuro-Cranio-Vertebral Syndrome*.*

## Methods

Between the 14th of April, 2009 and 16th of December, 2015, 1285 patients with one or several of the diagnoses of Arnold-Chiari Syndrome Type I, Idiopathic Syringomyelia, Idiopathic Scoliosis, Platybasia, Basilar Impression, Brainstem Kinking, low-lying conus medullaris and related pathologies were seen at the *Institut Chiari & Siringomielia & Escoliosis de Barcelona* out of whom, we present the clinical and imaging features in a sample of 373 patients, selected because they presented complete data registered for the purposes of this investigation, after excluding cases with significant neurological or neurosurgical antecedents that could interfere with their clinical or imaging presentation: procedures such as suboccipital craniectomy, syringostomy, ventriculo-peritoneal shunting, instrumentation for scoliosis, discectomies, laminectomies for spinal stenosis, as well as demyelinating, inflammatory, tumorous or traumatic diseases of the central and peripheral nervous system.

Patients generally contact us after having been diagnosed with one or more of these conditions in their home country, because of their interest in our method used for the diagnosis, treatment and follow-up of the Filum Disease and Neuro-Cranio-Vertebral Syndrome, called Filum System® (FS®, presented on https://filumsystem.com/enfermedad-del-filum, https://filumsystem.com/enfermedades-implicadas/ and https://institutchiaribcn.com) as we are the only center qualified to apply it worldwide, as a highly specialized private center holding the Research & Development (R&D) certification 1583.001.16–160,920-CER-RD.001 from the Spanish Innovation Certification Agency (ACIE) and ENAC certification 33/C-PR074, Certificate IQNet and AENOR Quality Management System ISO 9001:2015, Registration Number: ES-0081/2015 for the following fields of activities: Research, Diagnosis and Treatment of the Filum Disease and Quality Management Certification according to UNE-EN ISO 9001:2008 standards.

**Fig. 1:**
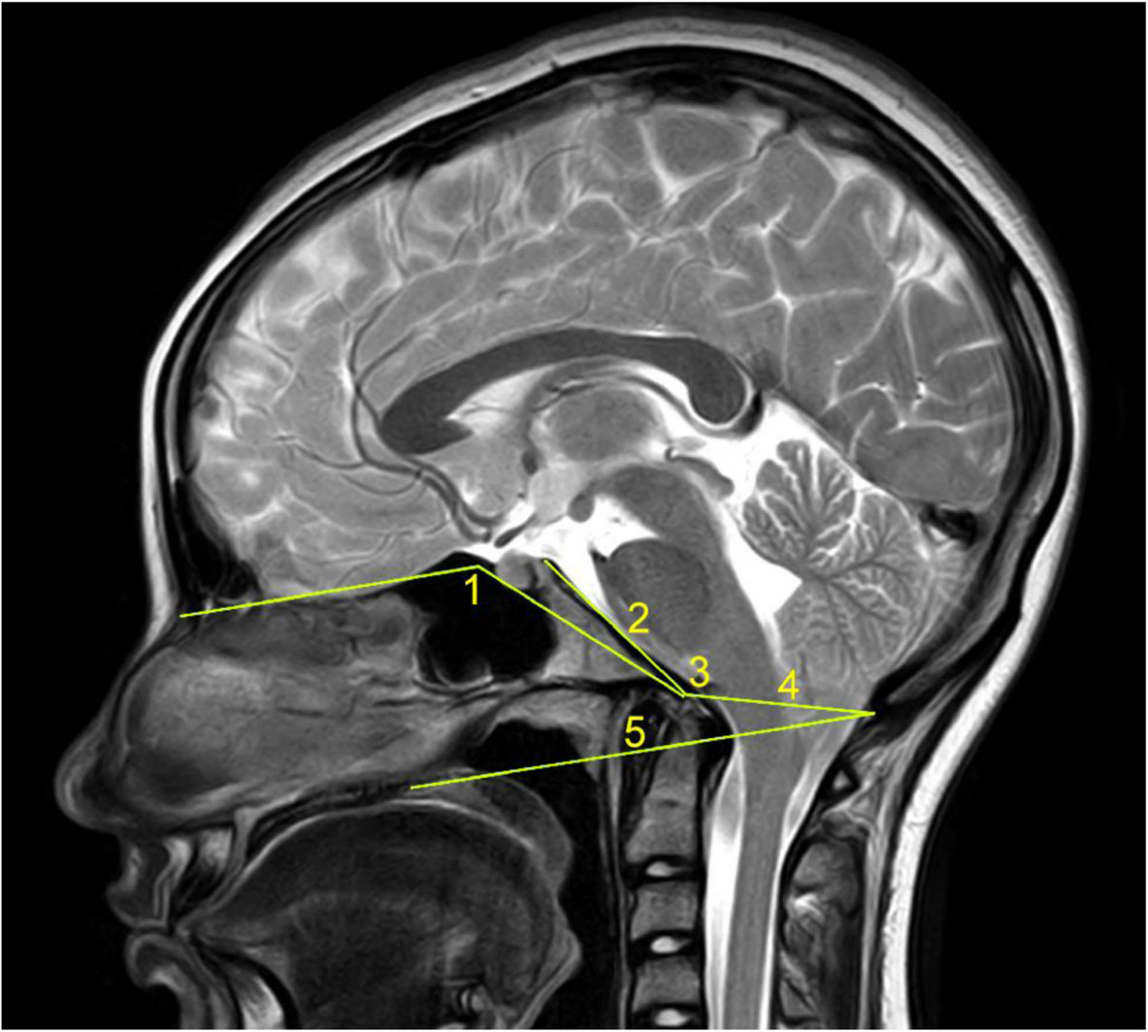
Parameters employed in the assessment of Occipitocervical Junction Malformations: 1 – Welcher’s basal angle; 2 - Thiébaut-Wackenheim-Vrousos’ basilar line; 3 – Boogaard’s angle; 4 – McRae’s line; 5 - Chamberlain’s line

Once the patients have arrived and have been registered at our center, after recording personal and family antecedents, the history focuses on a meticulous interview about possible Neuro-Cranio-Vertebral Syndrome symptoms, according to an anatomical order, followed by an exhaustive and detailed neurological examination centered on the Neuro-Cranio-Vertebral Syndrome and consisting mainly of the procedures presented in Table 1. The majority of patients send magnetic resonance imaging (MRI) series of the whole spine previous to their appointment, including at least sagittal and axial cuts, T1 as well as T2-weighted, as well as antero-posterior and lateral x-ray images of the entire spine taken in standing position. All these are scrutinized in the search for the following pathologies:

1. Occipitocervical Junction Malformations, of which the most common are: Basilar Impression with the odontoid ascending more than 5 mm over Chamberlain’s line; Platybasia with an increased Boogaard’s angle of more than 135° or a Welcher’s basal angle of more than 140°; Retroflexed Odontoid with an inclination of the dens more than 2 mm behind the prolongation of the basilar line of Thiébaut-Wackenheim-Vrousos; Brainstem Kinking as seen in significant cases of Platybasia (Fig. 1).

**Table 1:** Neurological examination. ^1^Currently, we also employ the Jamar dynamometer.^2^ Not included in the statistical analysis

Procedures		Findings
1.	Pupillary examination	Miosis, mydriasis, anisocoria, diminished reflex to light
2.	Oculomotricity	Strabismus, nystagmus
3.	Inspection uvula and tongue	Deviation, asymmetry
4.	Grip strength measurement with Collins dynamometer^1^	Unilateral or bilateral decrease below the 10th centile of the corresponding age and gender group
5.	Deep tendon reflexes, abdominal reflexes and plantar reflexes	absent, decreased, brisk, appearance of pathological reflexes
6.	Sensibility to temperature in at least 40 body areas	Anesthesia, hypoesthesia, hyperesthesia, dysesthesias or evoked paresthesias
7.	Sensibility to touch in at least 40 body areas	Anesthesia, hypoesthesia, hyperesthesia, dysesthesias or evoked paresthesias
8.	Lasègue’s test	Positive if pain elicited, specifying its location and leg elevation angle
9.	Mingazzini’s test	Paresis
10.	Reversed Lasègue’s test^2^	Positive if thigh pain elicited with flexion of the leg in prone position
11.	Barré’s test^2^	Paresis
12.	Tender points on the back and lower limbs ^2^	Positive if pain elicited in certain spots with thumb pressure
13.	Inspection and palpation of sacral area	Deformation, sacral dimple, hypersensitivity
14.	Inspection of back, shoulders and scapulae^2^	Shoulder asymmetry, winged scapulae waist fold sign thorax, scoliotic attitude
15.	Romberg’s test	Instability, retropulsion or lateropulsion
16.	Toe and heel walking^2^	Paresis, instability, ataxia
17.	Quadriceps paresis test ^2^	Positive if difficult or impossible to stand up from alternate unilateral kneeling position

**Fig. 2:**
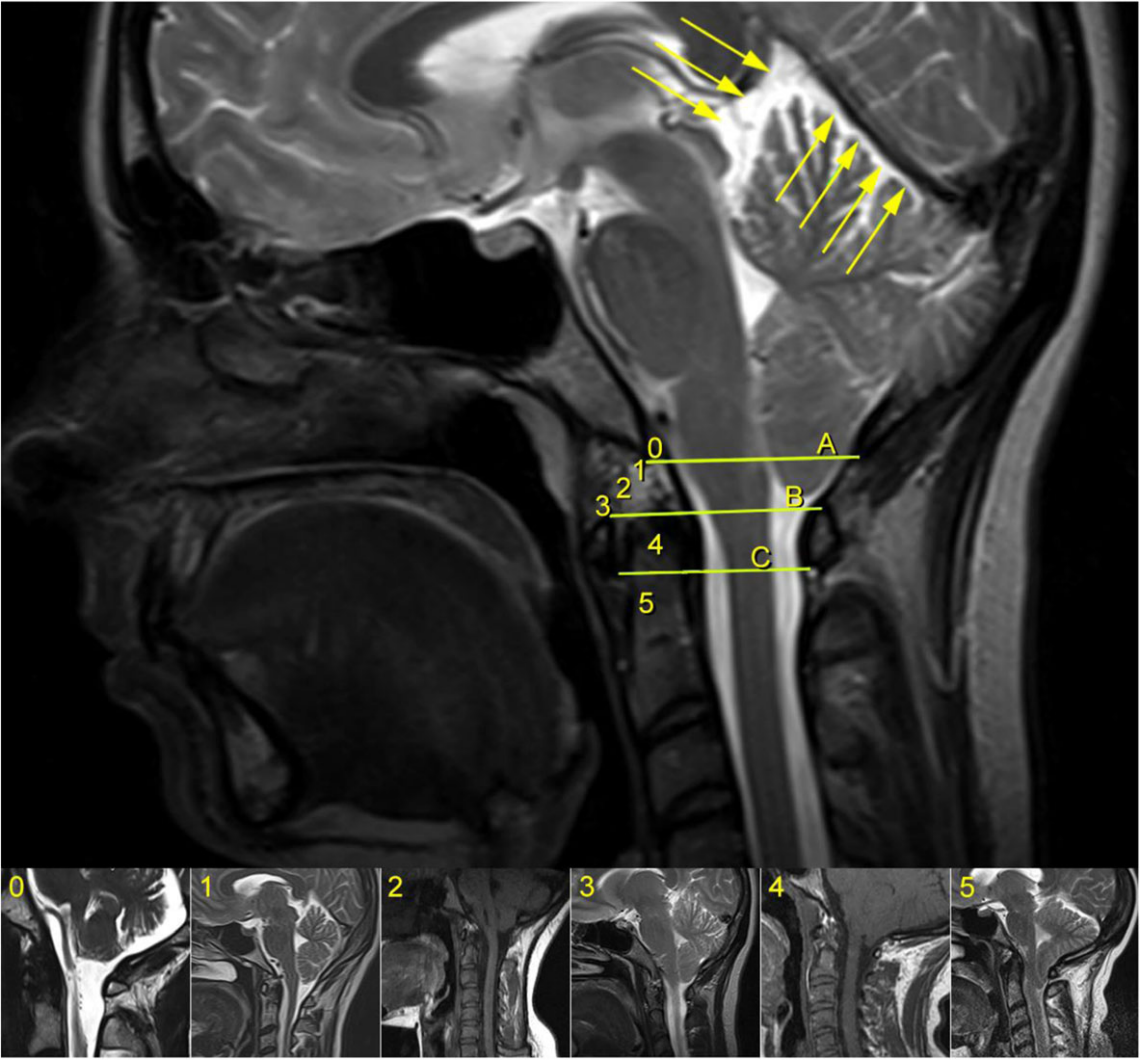
Classification of the magnitude of DCT with examples for each degree. A – McRae’s line (FM); B – Upper border of atlas (C1); C – Lower border of atlas (C1). The interval A-B has been divided into an upper (grade 1), middle (grade 2) and lower (grade 3) third. They are followed by grade 4 – between the upper and lower borders of C1, and finally grade 5 – Underneath the lower border of C1. When the tonsil reaches exactly line A, it is considered grade 0, that we call *Impaction of Cerebellar Tonsils*. The arrows indicate another relevant parameter often associated to DCT, *the Increase of the Supracerebellar Space*

Descent of Cerebellar Tonsils (DCT), defined as any descent of one or both cerebellar tonsils underneath the foramen magnum plane, represented by McRae’s line. Furthermore, instead of measuring the length of the displacement in millimeters as is customary, we report it in relation to the occipitocervical bony structures reached by the tip of the lowermost tonsil, like the foramen magnum, the posterior arch of the atlas (C1) and the spinous process of the axis (C2) (Fig. 2). We also define as Impaction of Cerebellar Tonsils their contact or intimate closeness with McRae’s line, that we consider to be an incipient form of Descent of the Cerebellar Tonsils, representing the equivalent of what other authors have denominated “Chiari malformation type 0”.

**Fig. 3:**
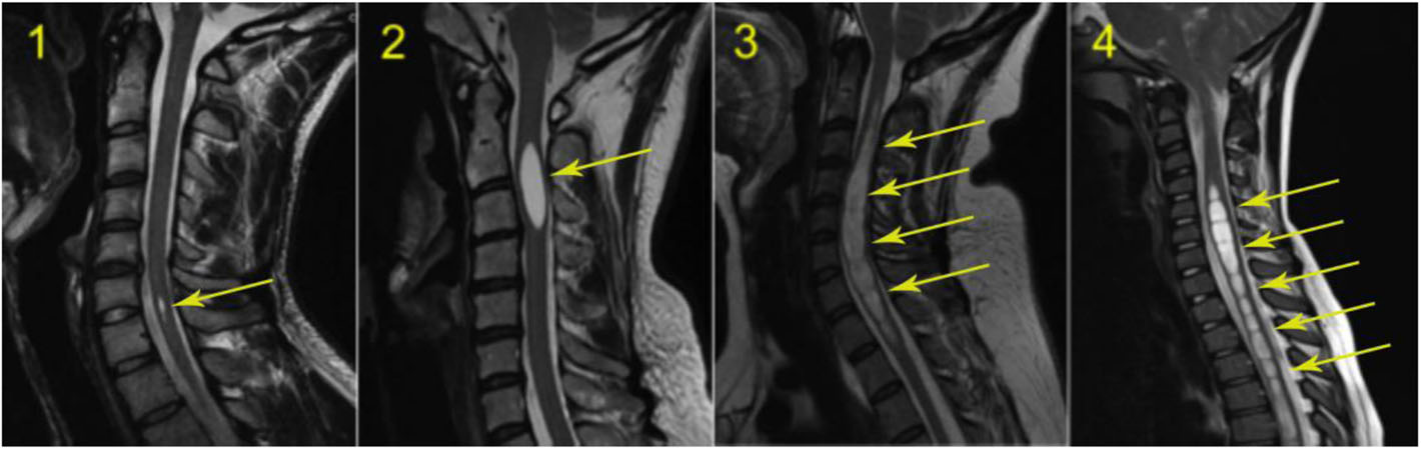
Classification of the extension of syringomyelic cavities Grade 1 – less than one vertebral segment; grade 2 - between 1 and 5 vertebral segments; grade 3 - between 6 and 10 vertebral segments; grade 4 – more than 10 vertebral segments

**Fig. 4:**
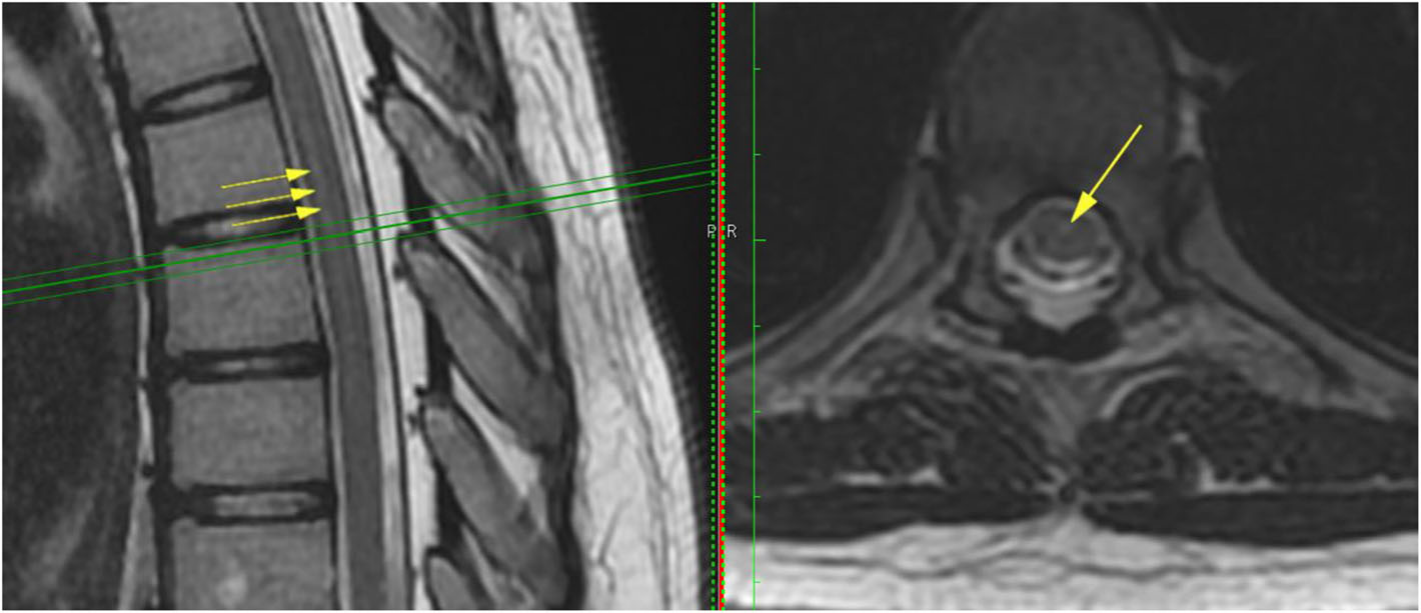
Spinal Cord Ischemia-Edema visible in a portion of the thoracic spinal cord on the sagittal cut (left, arrows), corresponding with an image of centro-medullary edema on the axial cut (right, arrow)

**Fig. 5:**
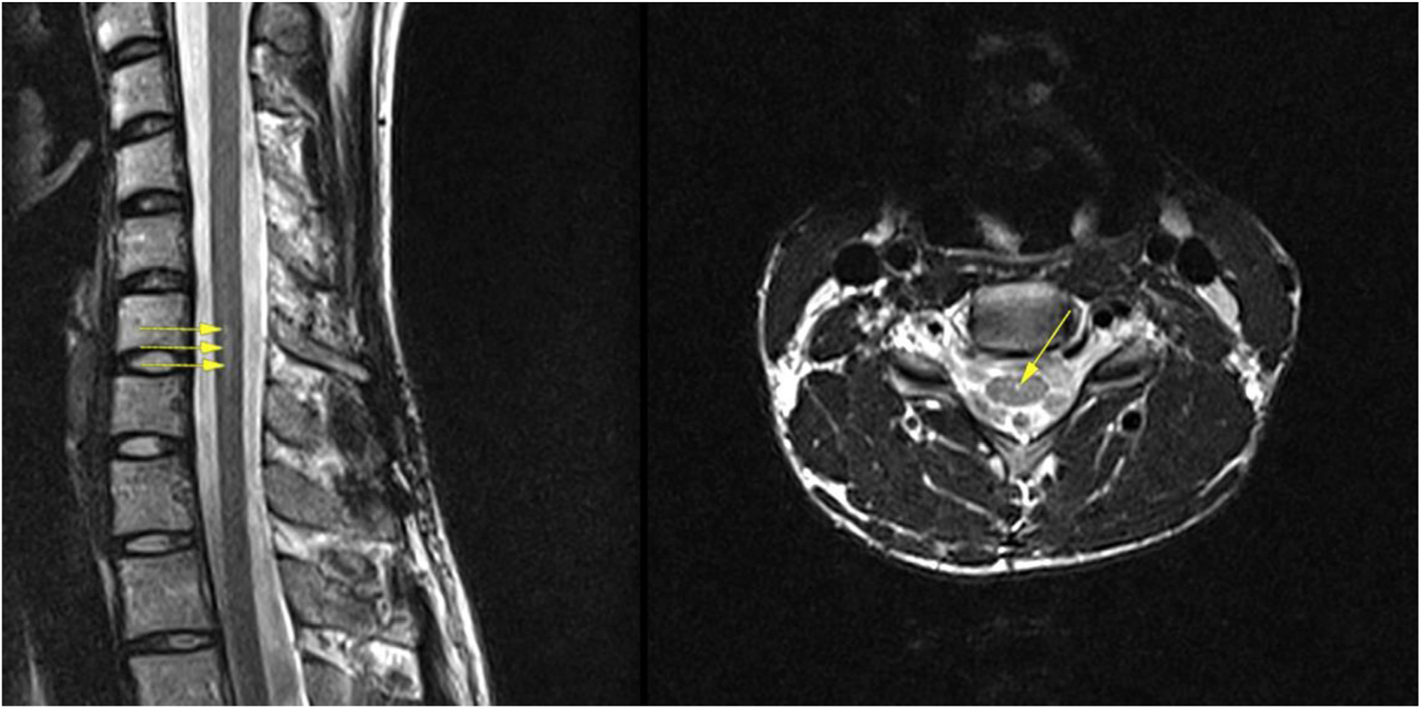
Central canal dilatation (arrows). There is also a nicely outlined Tense Spinal Cord on the sagittal cut (left)

**Fig. 6:**
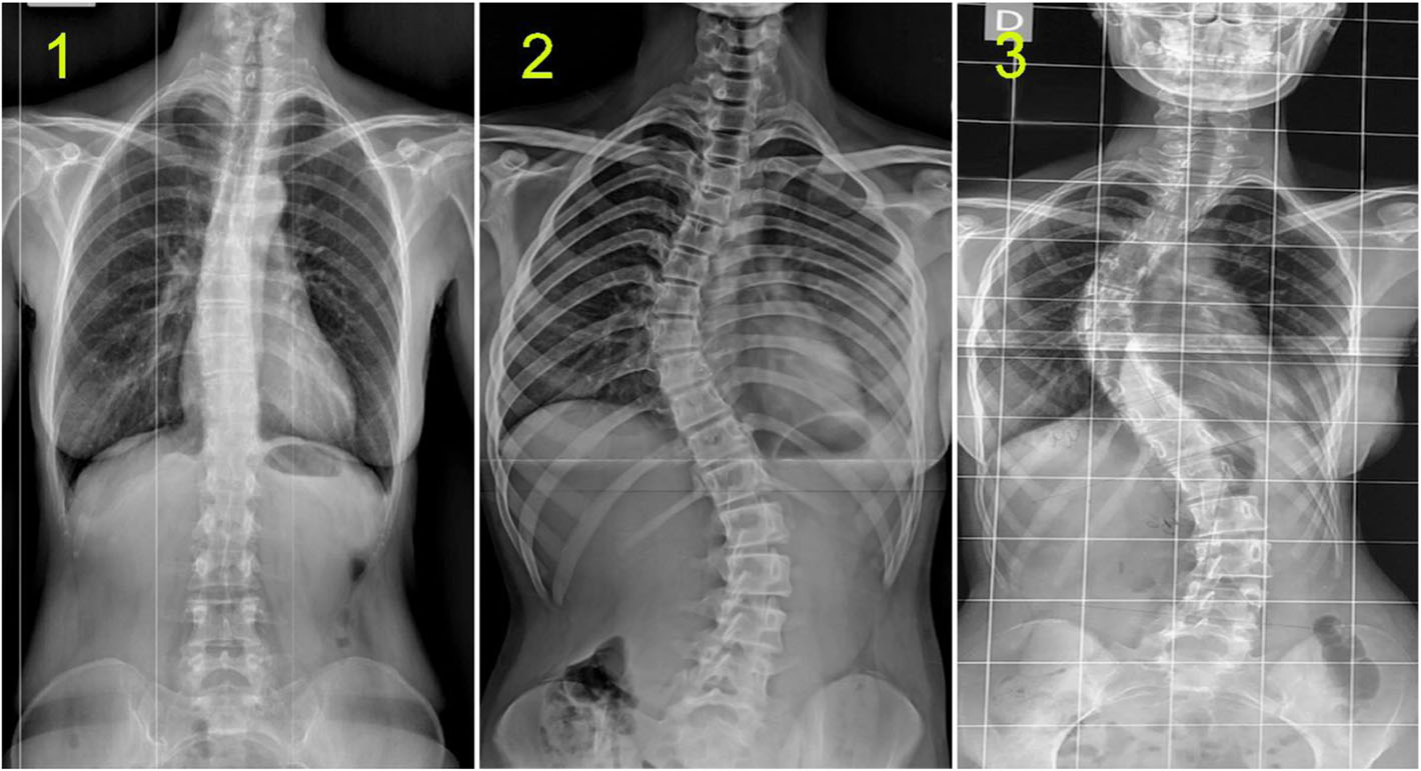
Classification of Idiopathic Scoliosis. 1 - mild; 2 - modrate; 3 – severe

**Fig. 7:**
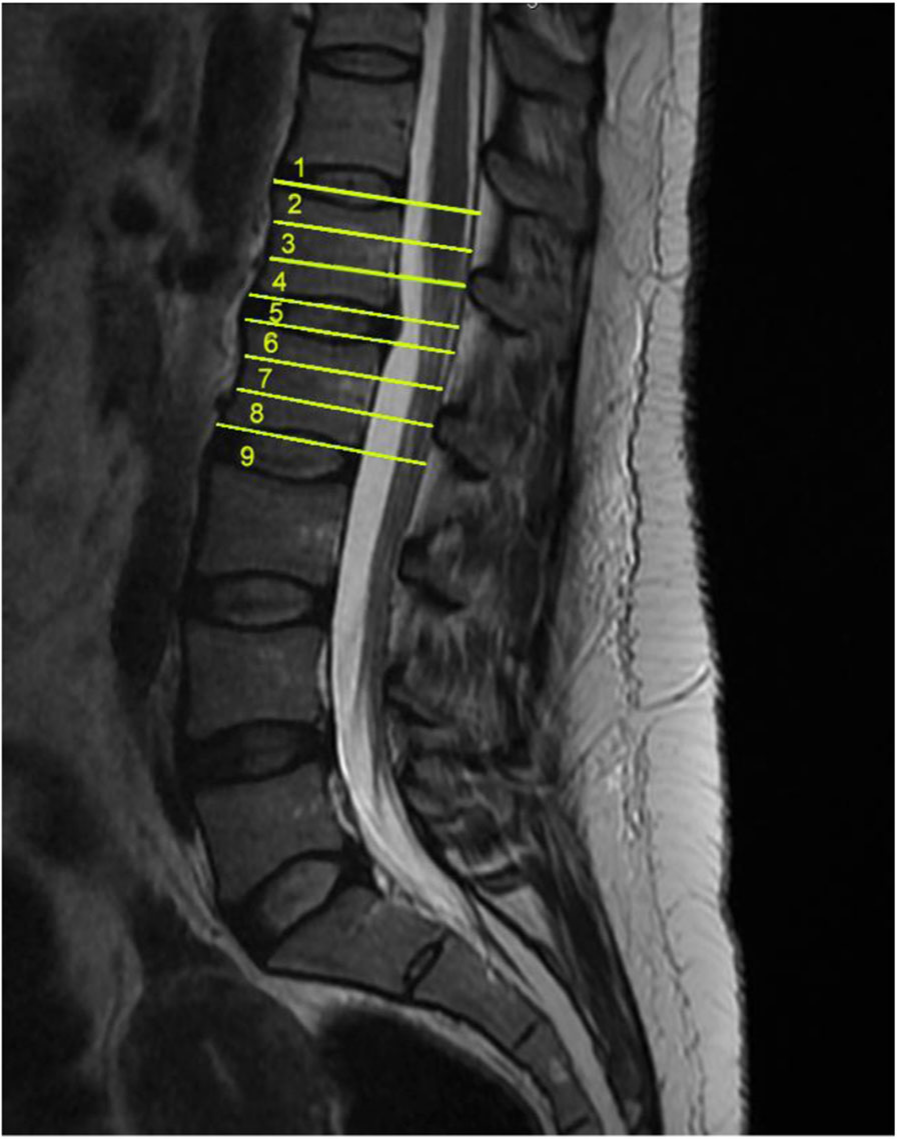
Levels used in the classification of the depth of the conus medullaris position with regard to vertebral segments: 1 - up to the D12L1 intervertebral disc; 2 - upper third of L1 vertebral body; 3 – middle third of L1 body; 4 – lower third of L1 body; 5 - L1L2 intervertebral disc; 6 - upper third of L2 body; 7 – middle third of L2 body; 8 – lower third of L2 body; 9 –lower than L2 body

2. Intramedullary Cyst (IMC), defined as an idiopathic syringomyelic cavity of any size, shape and location, always having ruled out a tumoral, vascular or inflammatory etiology by the absence of abnormal contrast enhancement, as well as a traumatic etiology by patient’s history and absence of any associated traumatic injuries on imaging. Moreover, cavities were classified according to the longitudinal extension along one or more vertebral levels (Fig. 3). We also take into consideration the following two types of presyringomyelic lesions: a) Spinal Cord Ischemia-Edema, defined by an aspect of one or two parallel hyperintense lines within the spinal cord on T2-weighted sagittal images, usually associated with visible medullary edema (as focal hyperintensity) on T2-weighted axial images (Fig. 4) [29] and b) Central Canal Dilatation, when this one becomes visible, but without reaching the diameter of a filiform syringomyelic cavity (Fig. 5).
3. Deviation of the Vertebral Column (DVC) or Idiopathic Scoliosis defined as any coronal plane disalignment resulting in scoliotic curvatures and being detectable on the standing spine x-ray, that we classify into the following three categories: mild, if it is up to 10° calculated with the Cobb method; moderate, if it measures between 10 and 40° Cobb and severe if it is greater than 40° Cobb (Fig. 6). We consider the presence of any abnormality of sagittal spinal curves equally significant as the coronal curve, as proof of an abnormal cord tension, from straightening to inversion of the “physiological” sagittal curvature of any vertebral region.
4. Low-lying Conus Medullaris (LCM), defined as a position of the tip of the medullary conus lower than the D12L1 intervertebral disc, classified according to the corresponding vertebral segment where it reaches, each vertebral body being divided in thirds (Fig. 7).

**Fig. 8:**
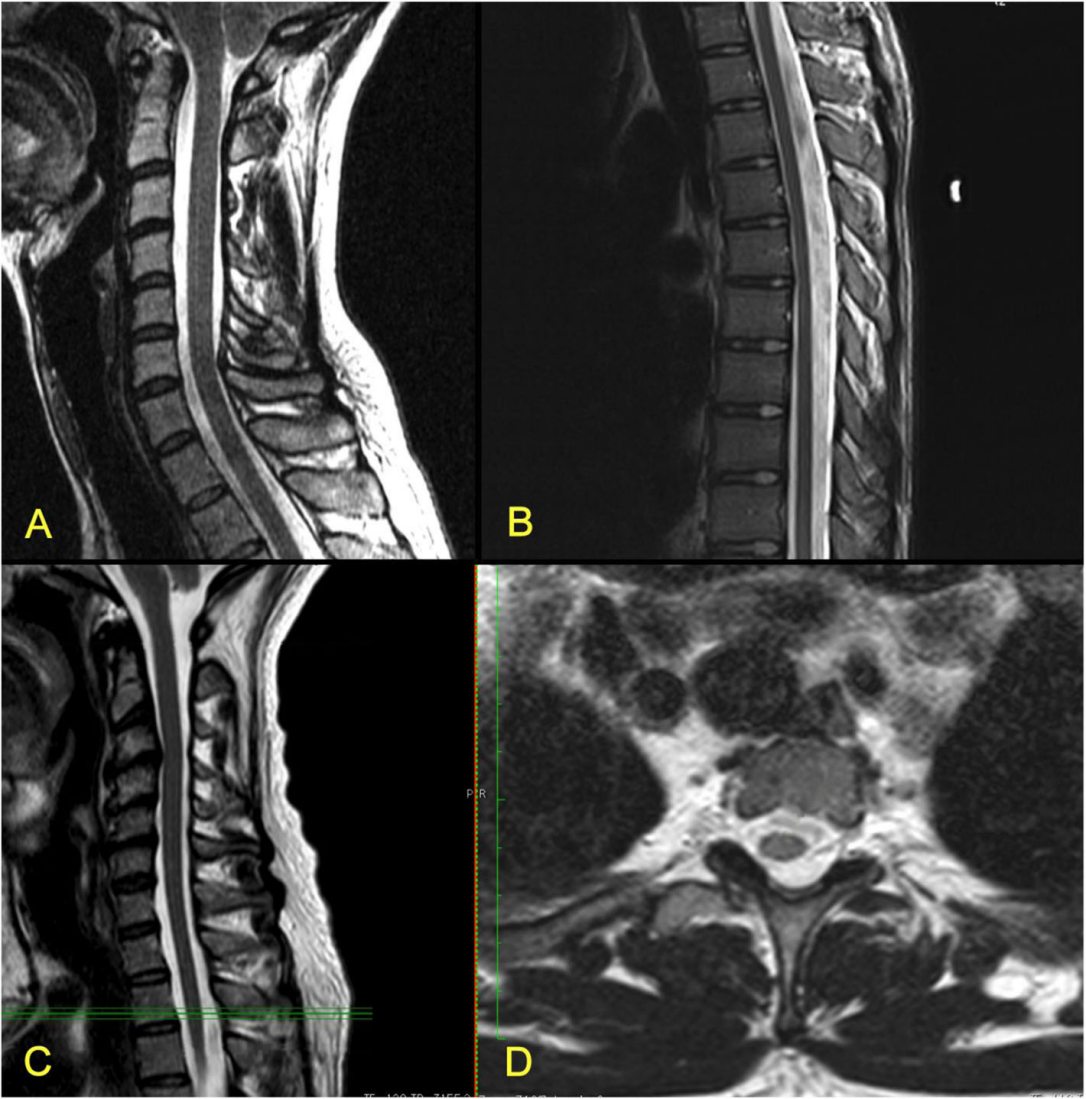
MRI cuts showing features of Tense Spinal Cord (A, C - cervical spine; B - thoracic spine) and Lateralized Spinal Cord (D, at the level marked with green lines in C)

We often observe as well other suggestive MRI features, for instance the Increase of the Supracerebellar Space (Fig. 2), the Tense Spinal Cord (in sagittal cuts, Figs. 5 and 8) and Lateralized Spinal Cord (in coronal or axial cuts, Fig. 8), a visible *Filum terminale internum* and/or *externum* and finally, rotoscoliosis; however, despite being frequent, these changes have not been subject of the statistical analysis in this patient group.

Digital images that came in JPEG format were visualized with the program Preview version 8.1 (Apple, Inc. Cupertino, CA, USA), whilst the majority of them, in DICOM format, were viewed with the program OsiriX version 5.8.2 (Pixmeo SARL, Bernex, Switzerland).

For data analysis, general data, clinical symptoms, clinical signs and imaging features were recorded during the visit of every patient in a table included in a digital database (FileMaker Pro Advanced 11.0v2, FileMaker, Inc. Santa Clara, CA, USA), from where they were transferred to a Microsoft Excel 2011 spreadsheet for Mac version 14.1.0 (Microsoft Corporation, Redmond, WA, USA) and then into an SPPS database (version 21, IBM Corporation, Armonk, NY, USA).

We performed an initial descriptive analysis of the general data (gender, age, type and duration of clinical course), dividing the variables into three main categories: clinical symptoms, clinical signs and imaging features. The variables followed in this study were analyzed looking for associations between components of the three categories mentioned above, taking into account topographical criteria. We used Pearson’s chi-squared test and Kendall’s test for ordinal data and Mantel-Haenszel chi-square test for stratified data, considering *p* < 0.05 as significance threshold. Finally, we created continuous variables by grouping symptoms and signs according to topographical criteria (Table 2) and these variables, as well as the different kinds of imaging features were analyzed together, first through comparison of the means and Student’s *t*-test for independent samples and then through the creation of dispersion diagrams and computing Pearson’s correlation coefficient.

**Table 2:** Creation of new continuous scale variables – for each given patient, the value of the variable was the sum of the individual values of the different categorical variables, selected according to topographical criteria

New continuous variable	Components	Range of values
General Symptoms	Cognitive deterioration+ Mood alterations + Insomnia + Fatigue	4–11
Cranial Symptoms	Headache + Nausea/vomiting + Balance alterations + Dysphagia + Visual alterations + Tinnitus + Diplopia	7–15
Cervical Symptoms	Cervical pain + Upper limb pain + Upper limb numbness + Upper limb motor loss	4–8
Spinal Cord Symptoms	Thoracic back pain + Low back pain + Lower limb pain + Thoracic pain + Lower limb numbness + Lower limb motor loss + Paresthesias + Alterations of temperature perception + Muscular cramps + Sphincter alterations + Abnormal gait	11–21
Cranial Signs	Nystagmus + Deviation of the uvula and/or tongue	2–4
Medullary Signs	Alterations of temperature sensibility + Alterations of sensibility to touch + Upper limb deep tendon reflexes + Lower limb deep tendon reflexes + Abdominal reflexes + Plantar reflexes + Lasègue’s test + Mingazzini’s test+ Romberg’s test+ Grip strength	10–25

## Results

### Part I – descriptive analysis

#### General data

**Fig. 9:**
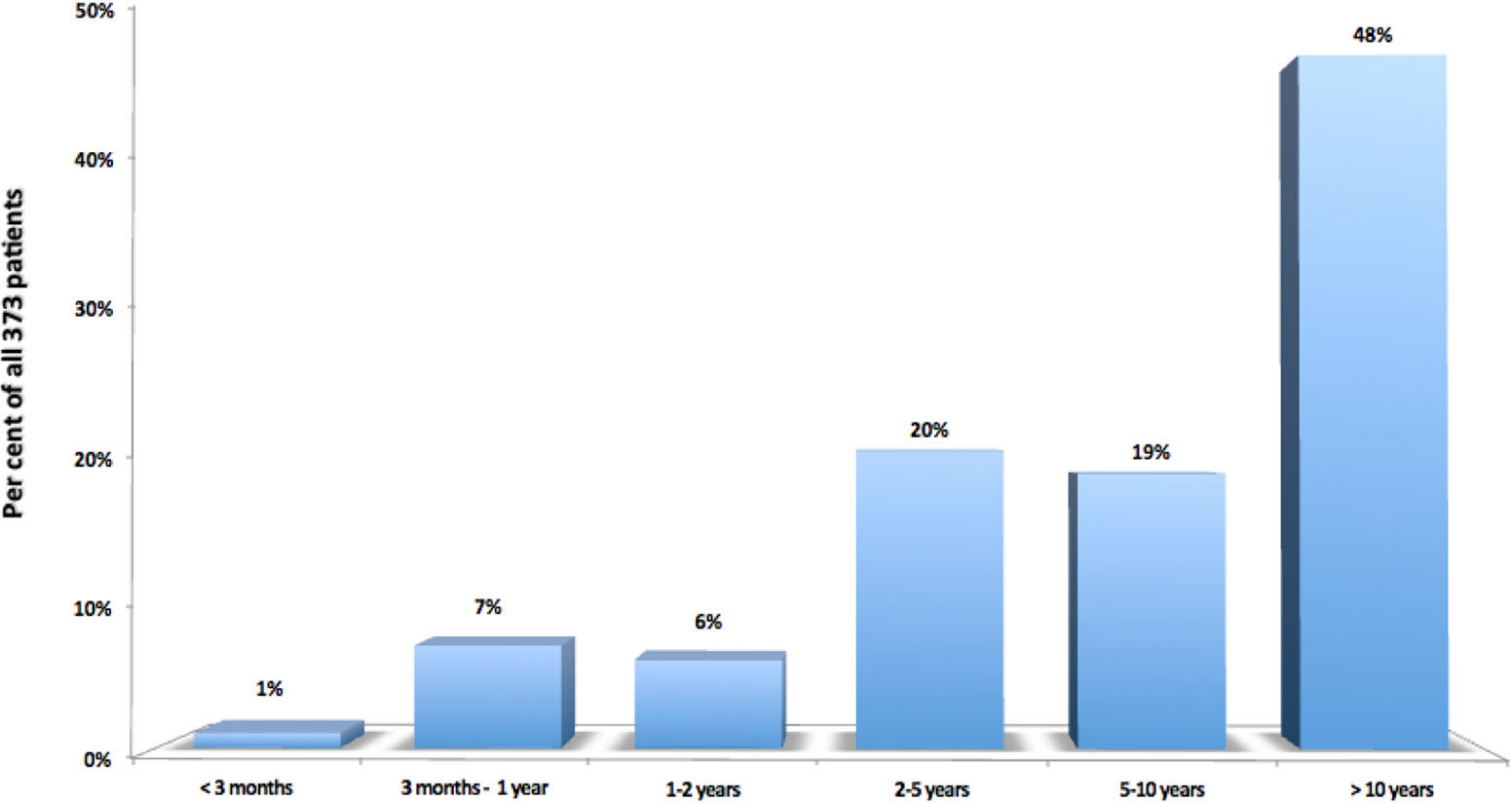
Time interval from initiation of first symptoms until the diagnostic visit at ICSEB, in the selected 373 patients

Among the 373 selected patients, 270 were females (72%), with ages between 3 and 76 years (median 33, mean 33.66, standard deviation 15.87). The time interval from the appearance of the first symptoms until the establishment of the diagnosis was greater than 10 years in 177 cases (48%), between 5 and 10 years in 70 cases (19%), between 2 and 5 years in 76 cases (20%) and rarely shorter (Fig. 9).

#### Neurological clinical picture

The symptoms detected in more than 10% of analyzed patients and the clinical signs detected through the specific neurological examination are presented in Tables 3 and 4.

**Table 3:** Frequency of clinical symptoms in the selected 373 patients. ^1^Instability, dizziness, vertigo, etc. ^2^Blurred vision, phosphenes, scotomata, etc. ^3^Sensation of cold hands and/or feet, intolerance/unawareness of coldness/warmth. ^4^Incontinence/retention, urgency, frequency, etc. ^A^Later during the analysis, grouped together as “Cognitive deterioration”. ^B^Later during the analysis, grouped together as “Mood alterations”

Symptom		Frequency	Percentage
1.	Headache	312	84
2.	Nausea and/or vomiting	182	49
3.	Balance alterations^1^	268	72
4.	Difficulty swallowing	141	38
5.	Visual alterations^2^	212	57
6.	Double vision	58	16
7.	Tinnitus	171	46
8.	Speech disorders^A^	92	25
9.	Memory deterioration^A^	162	43
10.	Attention alteration^A^	143	38
11.	Sadness^B^	100	27
12.	Anxiety^B^	45	12
13.	Nervousness^B^	196	53
14.	Sleeplessness	181	49
15.	Fatigue	183	49
16.	Upper extremity pain	167	45
17.	Lower extremity pain	208	56
18.	Thoracic pain	81	22
19.	Cervical pain	268	72
20.	Thoracic back pain	243	65
21.	Lumbosacral pain	270	72
22.	Upper extremity numbness	110	30
23.	Lower extremity numbness	76	20
24.	Paresthesias	262	70
25.	Alterations of temperature perception^3^	146	39
26.	Cramps	44	12
27.	Upper extremity weakness	182	49
28.	Lower extremity weakness	175	47
29.	Sphincter alterations^4^	192	52
30.	Gait alteration	170	46

**Table 4:** Frequency of clinical signs in the selected 373 patients

Signs		Frequency	Percentage
1.	Spontaneous nystagmus	204	55
2.	Deviation of uvula and/or tongue	237	64
3.	Altered sensibility to temperature	257	69
4.	Altered sensibility to touch	242	65
5.	Altered deep tendon reflexes in upper extremities	322	86
6.	Altered deep tendon reflexes in lower extremities	309	83
7.	Altered cutaneous abdominal reflexes	254	68
8.	Altered cutaneous plantar reflexes^1^	274	73
9.	Positive straight leg-raising test	165	44
10.	Positive Mingazzini’s test	245	66
11.	Positive Romberg’s test	188	50
12.	Decreased grip strength	259	70

^1^Babinski’s sign was present on one or both sides in 109 patients (29%)

It is worthwhile to mention that there were other symptoms as well, that we observed quite frequently throughout the study, but as they had not been contemplated in the initial list, they were not assessed in the present analysis and we can only give an overall count of the presence of each one in this patient sample: photophobia in 137 cases (37%), sonophobia in 126 cases (34%), involuntary movements or fasciculations in various body parts in 57 cases (15%) and sensations of electrical current in different regions in 40 cases (11%).

On the contrary, other symptoms, although included in the study from the start, were observed too rarely to be used in the actual analysis and were therefore rejected as not being specific enough for this clinical picture: loss of consciousness in 23 cases (6%), dysphonia in 20 (5%), hypersomnia in 20 (5%), abdominal pain in 24 (6%), dysesthesias in 28 (8%), tremor in 29 (8%) and atrophy of various body segments in 22 (6%). It is furthermore notable that only 14 of our patients (4%) reported sleep apnea.

**Fig. 10:**
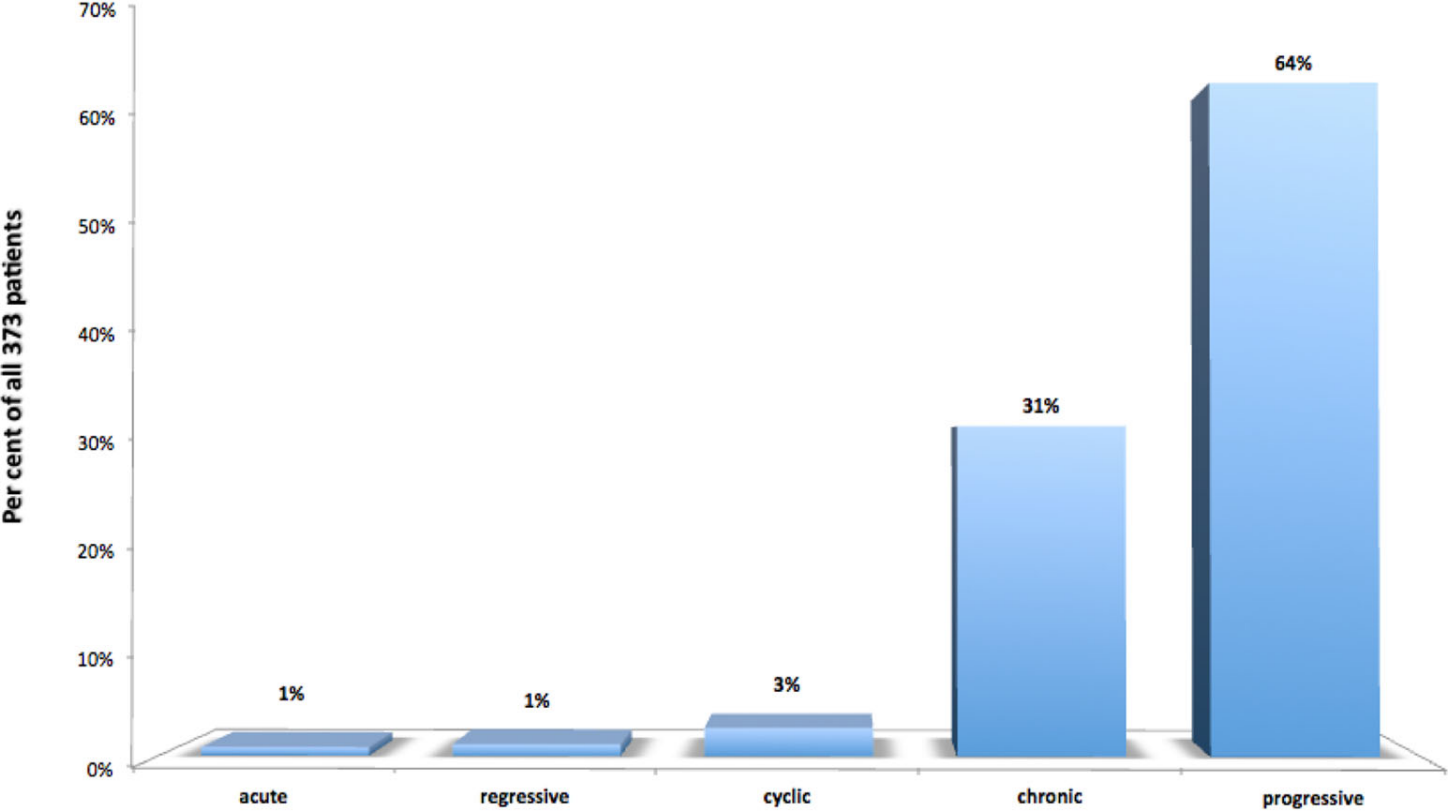
Type of clinical course in the selected 373 patients. *Acute* = symptoms appearing in the preceding 6 months; *regressive* = symptoms slowly diminishing or disappearing over time; *cyclic* = symptoms presenting in flare-ups separated by periods of normality; *chronic* = symptoms more or less constant over long periods of time; *progressive* = symptoms increasing in intensity and/or number over time

Concerning the type of clinical course, it was progressive in the majority of cases (239 patients, 64%), followed by a chronic type (177 patients, representing 31%); the remaining were much less frequent (Fig. 10).

#### Imaging features

**Fig. 11:**
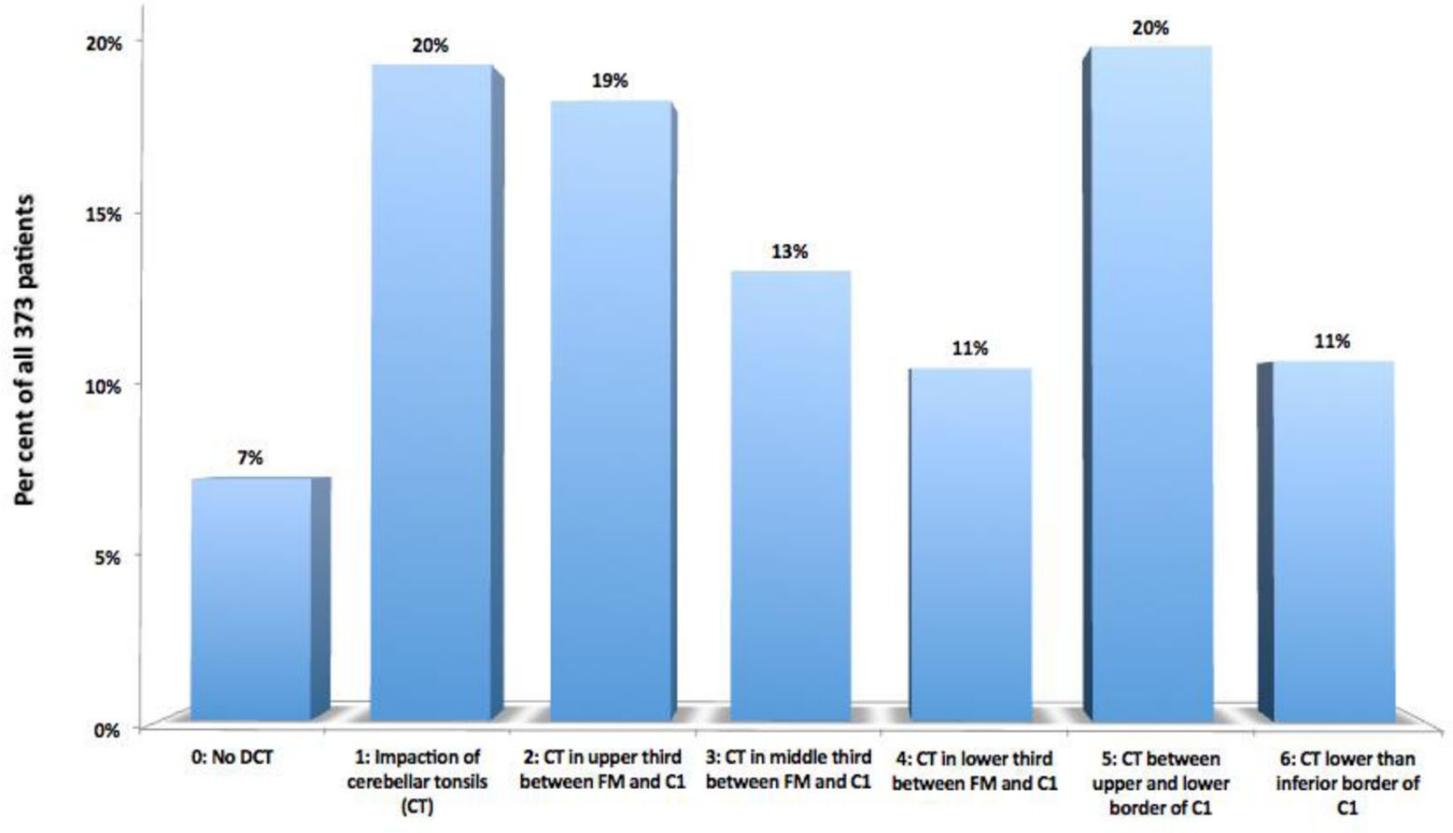
Descent of the cerebellar tonsils (DCT) in relation to FM, posterior arch of C1 and C2, in the selected 373 patients. CT = cerebellar tonsils; FM = foramen magnum; C1 = first cervical vertebra

**The Descent of the Cerebellar Tonsils (Arnold-Chiari Syndrome Type I)** was present is 273 cases (73%), while other 73 cases (20%) were interpreted as **Impaction of the Cerebellar Tonsils**. We have found all degrees of descent in quite balanced proportions; the most frequent variant was the one with the tonsils reaching just in front of the posterior arch of the atlas (Grade 4 in Fig. 2) (75 cases, 20%) (Fig. 11).

**Fig. 12:**
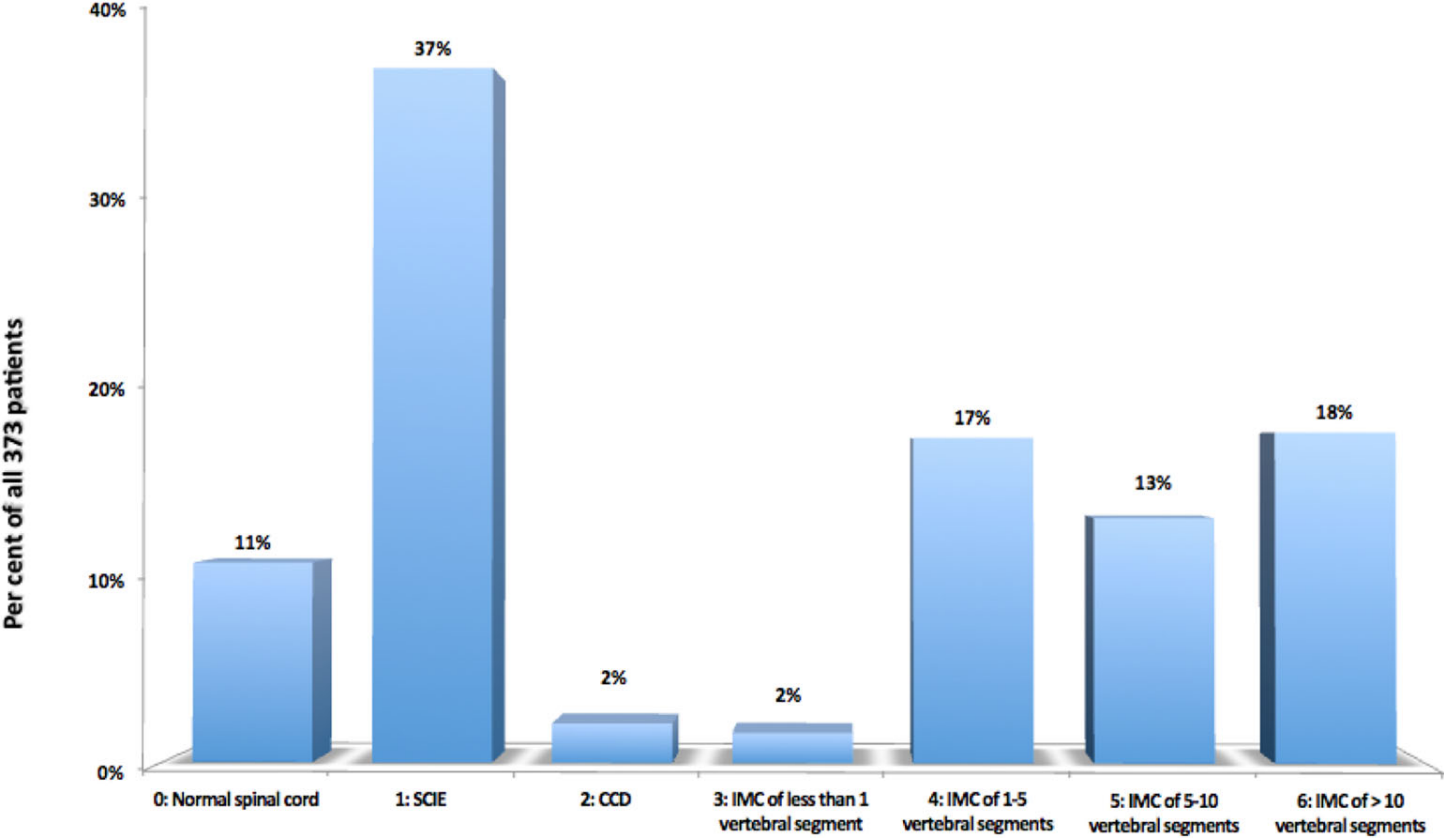
Frequency of presyringomyelic lesions and of the various degrees of longitudinal extension of syringomyelic cavities (Grade 0 corresponds to normal spinal cord), in the selected 373 patients. SCIE = Spinal Cord Ischemia-Edema; CCD = Central Canal Dilatation; IMC=Intramedullary Cyst

**Intramedullary Cysts (Idiopathic Syringomyelia)** were detected in 194 cases (52%), while 139 cases (37%) were interpreted as Spinal Cord Ischemia-Edema and another 8 cases (2%) had only a central canal dilatation. The most frequent location was cervicothoracic with 99 cases (26%) and it is worth noting that a cervical syringomyelia with or without variable extension in other vertebral segments was present in 135 cases (36%). As to the longitudinal extension of the syringomyelic cavity, most frequently it was over 10 vertebral segments (66 cases, 18%) (Fig. 12).

**Fig. 13:**
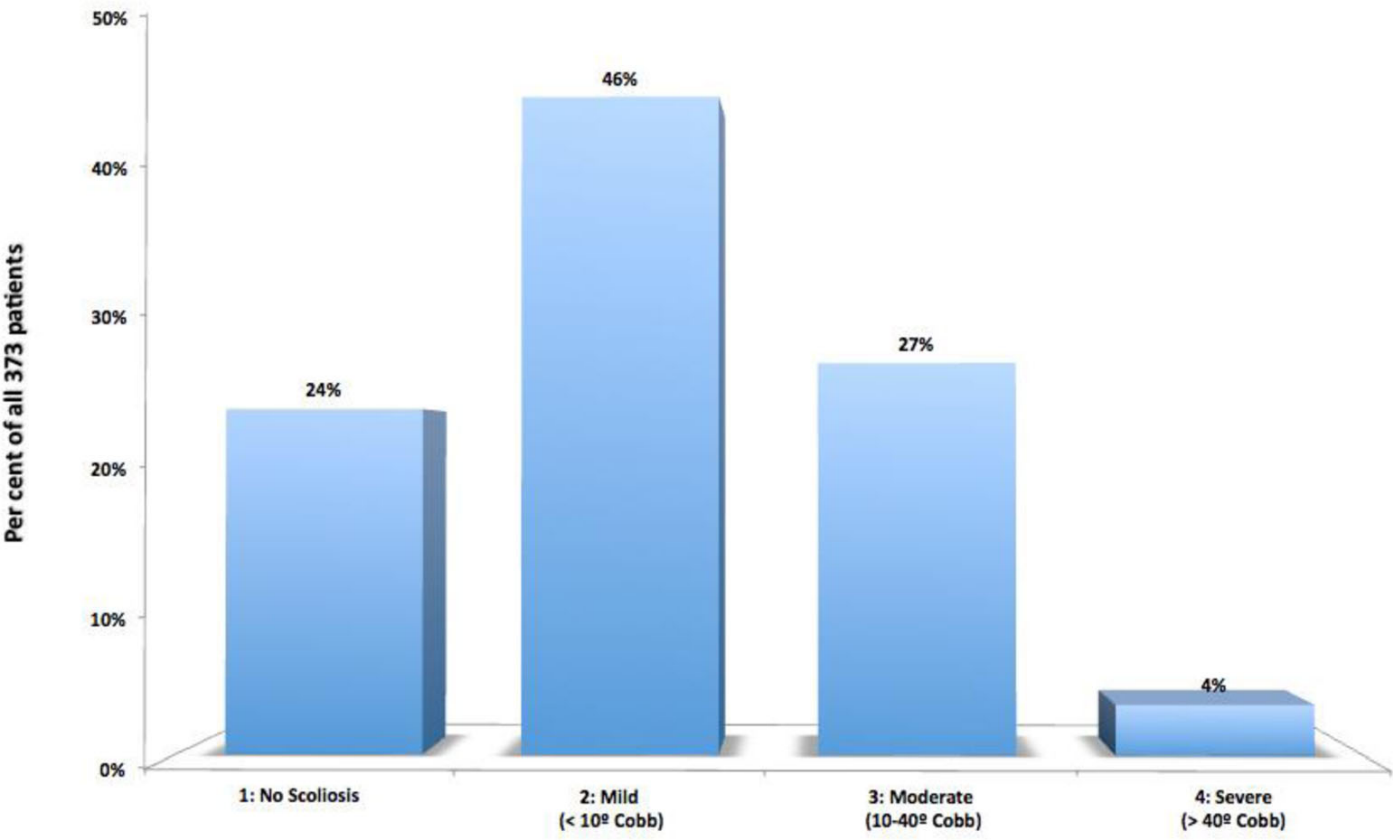
Presence of DVC (IS), classified by its severity measured according to Cobb’s method, in the selected 373 patients

**The Deviation of the Vertebral Column (Idiopathic Scoliosis)** was observed in 284 cases (76%), being mild (up to 10° Cobb) in the majority (170 cases, 46%) (Fig. 13).

**Fig. 14:**
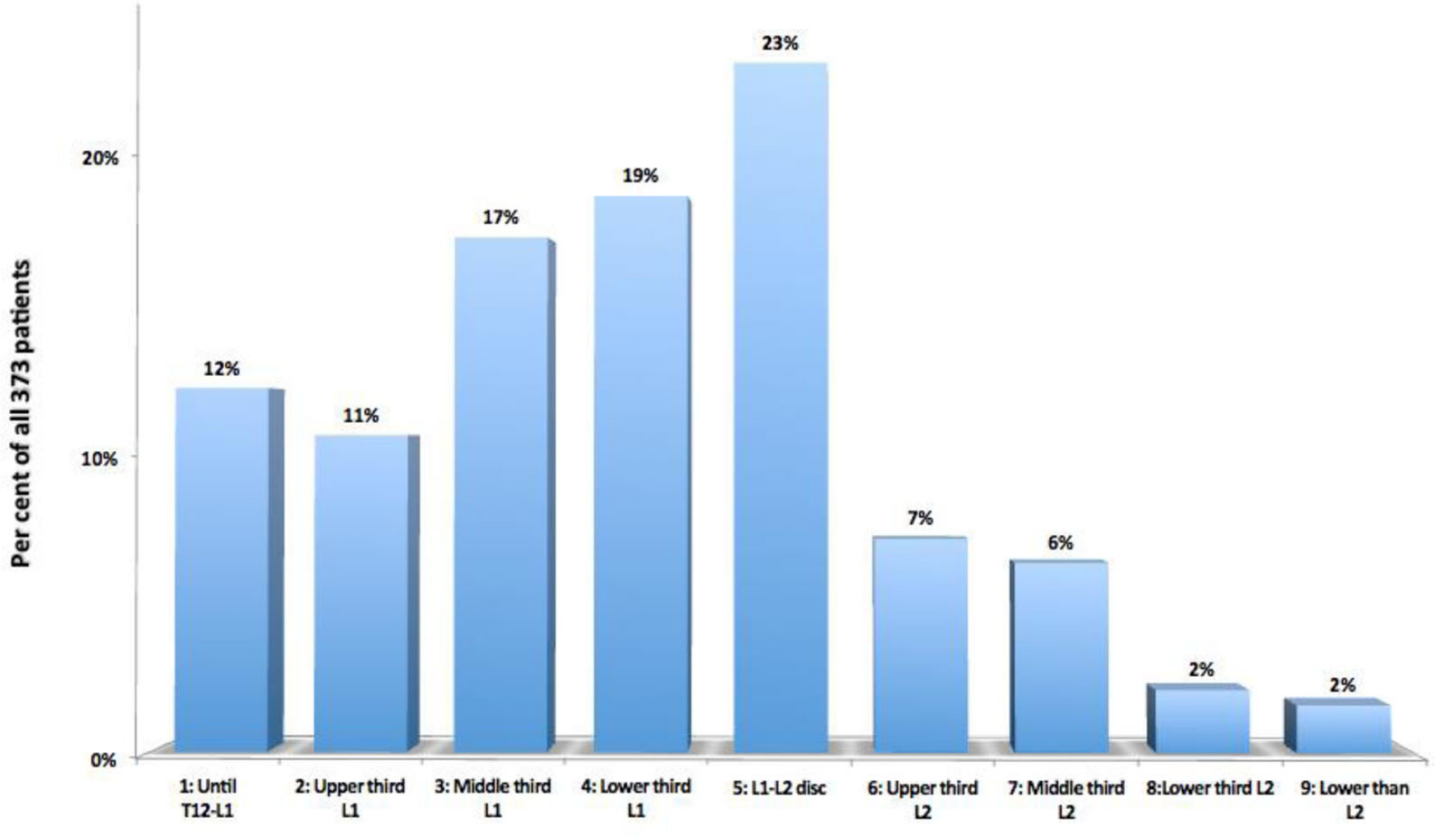
Height of the tip of the conus medullaris according to the corresponding vertebral levels (D12, L1, L2), from cranial to caudal, in the selected 373 patients

**The position of the tip of the conus medullaris** compared to the vertebral levels was very variable; the most frequent one was at the height of the L1L2 disc, found in 87 cases (23%) (Fig. 14).

As of **Occipitocervical Junction Malformations,** in this series we found 18 cases (5%) of **Retroflexed Odontoid (RO),** 15 cases (4%) of **Basilar Impression (BI),** 10 cases (3%) of **Platybasia (PTB) and** 6 cases (2%) of **Brainstem Kinking (BSK).**

Finally, a total of 267 cases (72%) were labeled as **Multiple-level Disc Disease**.

### Part II – bivariate analysis

The following statistically significant associations (*p* < 0.05) have been found:

A. CLINICAL SIGNS ASSOCIATED WITH CRANIAL OR GENERAL SYMPTOMS:

- **Deviation of the uvula and/or tongue** with: Headache (*p* = 0.011) and Nausea and/or vomiting (*p* = 0.014);
- **Spontaneous nystagmus** with: Balance alterations (*p* = 0.020 Kendall), Tinnitus (*p* = 0.000) and Cognitive deterioration (p = 0.011 Kendall);
- **Positive Romberg test** with: Balance alterations (*p* = 0.002);
- **Decreased grip strength** with: Cognitive deterioration (*p* = 0.021 Kendall);
- Altered sensibility to temperature with: Mood alterations (*p* = 0.000).

B. CLINICAL SIGNS ASSOCIATED WITH MEDULLARY SYMPTOMS:

- **Altered sensibility to temperature** with: Cervical pain (*p* = 0.004), Upper extremity pain (*p* = 0.000), Upper extremity numbness (*p* = 0.000), Upper extremity weakness (*p* = 0.000), Lower extremity pain (*p* = 0.045 Kendall) and Alterations of temperature perception (*p* = 0.000);
- **Altered sensibility to touch** with: Cervical pain (*p* = 0.044 Kendall), Upper extremity pain (*p* = 0.000), Upper extremity numbness (p = 0.000), Upper extremity weakness (*p* = 0.001) and Lower extremity pain (*p* = 0.011);
- **Alterations of deep tendon reflexes in upper extremities** with: Upper extremity weakness (*p* = 0.042) and Sphincter alterations (*p* = 0.024 Kendall);
- **Decreased grip strength** with: Upper extremity weakness (*p* = 0.000);
- **Alterations of deep tendon reflexes in lower extremities** with: Lower extremity weakness (*p* = 0.002) and Gait alteration (*p* = 0.046 Kendall);
- **Positive Mingazzini’s test** with: Lower extremity weakness (*p* = 0.001).

C. RELATIONSHIPS BETWEEN CLINICAL VARIABLES (SYMPTOMS AND SIGNS) AND IMAGING FEATURES:

- **Clinical variables associated with Descent of cerebellar tonsils:** Headache (*p* = 0.018), Deviation of the uvula and/or tongue (*p* = 0.013) and Decrease of grip strength (*p* = 0.042).
- **Clinical variables associated with Intramedullary cyst:** Nauseas and/or vomiting (*p* = 0.000), Visual alterations (*p* = 0.001), Tinnitus (*p* = 0.007), Cognitive deterioration (*p* = 0.001), Insomnia (*p* = 0.021), Fatigue (*p* = 0.000), Upper extremity numbness (*p* = 0.014), Alterations of temperature perception (*p* = 0.019), Upper extremity weakness (*p* = 0.006), Deviation of the uvula and/or tongue (*p* = 0.027), Altered sensibility to temperature (*p* = 0.001) and Altered cutaneous abdominal reflexes (*p* = 0.002). If we take into consideration only syringomyelias with cervical component, all of these variables continue to be associated, with the exception of insomnia, and there are also significant associations with: Upper extremity pain (*p* = 0.003), Lower extremity numbness (*p* = 0.045), Altered sensibility to touch (p = 0.000) and Altered cutaneous plantar reflexes (*p* = 0.010).
- **Clinical variables associated with Deviation of the vertebral column (Idiopathic scoliosis):** Thoracic back pain (*p* = 0.034), Spontaneous nystagmus (*p* = 0.038 Kendall), Altered sensibility to touch (*p* = 0.013), Altered cutaneous abdominal reflexes (*p* = 0.044 Kendall) and Altered cutaneous plantar reflexes (*p* = 0.001).

D. RELATIONSHIPS AMONG VARIOUS IMAGING FEATURES:

- **Descent of cerebellar tonsils** with: Occipitocervical junction malformations (*p* = 0.015 Kendall), Syringomyelia with cervical component (p = 0.003), Level of conus medullaris (*p* = 0.008) and Deviation of the vertebral column (*p* = 0.014 Kendall);
- **Level of conus medullaris** with Deviation of the vertebral column (*p* = 0.045 Kendall).

Moreover, through Mantel-Haenszel stratified analysis, it results that the positive relationship between the Descent of cerebellar tonsils and the Low-lying conus medullaris only exists in cases with moderate and severe scoliosis. It is also interesting that although seemingly there is no relationship between Descent of cerebellar tonsils and Intramedullary cyst when the latter includes all locations, in fact there is one when analyzed in detail, but the positive association between Descent of cerebellar tonsils and Syringomyelia with a cervical component is counterbalanced by a negative association of Descent of cerebellar tonsils with Syringomyelia with thoracic and/or lumbar locations.

The comparison of means and the *t*-test for independent samples, applied to the new continuous variables, formed by grouping symptoms and signs according to topographic criteria (Table 2) reveals the following positive relationships: Cervical symptoms – Syringomyelia with cervical component; Cranial symptoms - Descent of cerebellar tonsils; Cranial signs – Descent of cerebellar tonsils; and Medullary signs – Occipitocervical junction malformations.

It is worth mentioning that in all syringomyelias (intramedullary cysts), as well as if one considers only those with cervical component, there are less general and cranial symptoms than in patients without intramedullary cysts (they are negatively associated).

Concerning the Pearson correlation coefficient applied to the same new continuous variables, we observed good correlations within the group of clinical symptoms and signs – the strongest ones being between General symptoms – Cranial symptoms (r = 0.531, *p* = 0.000), Medullary symptoms – Medullary signs (r = 0.523, p = 0.000), Cervical symptoms – Medullary symptoms (r = 0.513, p = 0.000), Cranial symptoms – Medullary symptoms (r = 0.420, p = 0.000) and General symptoms – Medullary symptoms (r = 0.414, p = 0.000). There are statistically significant positive correlations (*p* < 0.05), nevertheless weaker (r between 0.106–0.149), between Cranial symptoms - Descent of cerebellar tonsils, Cranial signs - Descent of cerebellar tonsils, Cervical symptoms - Intramedullary cysts and Medullary symptoms - Intramedullary cysts. Similarly, there are weak statistically significant negative correlations (p < 0.05, r between 0.120–0.197) between General symptoms - Deviation of the vertebral column, General symptoms - Intramedullary cysts, Cranial symptoms -Intramedullary cysts, Medullary symptoms - Descent of cerebellar tonsils and Cranial signs - Intramedullary cysts. Generally, it is noteworthy that Descent of cerebellar tonsils has positive correlations with cranial symptoms and signs, while the second main image abnormality, Intramedullary cysts, has positive correlations with cervical symptoms and medullary signs.

## Discussion

Historically, the mentioned pathologies have been defined in general with one or two publications, like Fuchs in 1910 [3] and Lichtenstein in 1940 [4] for the tethered cord syndrome; Hoffman in 1976 [7] for occult tethered cord and Garceau in 1953 [5], for the cord-traction syndrome and filum terminale syndrome. In none of the above was any correlation demonstrated between Idiopathic Syringomyelia, Arnold-Chiari Syndrome Type I, Idiopathic Scoliosis, Platybasia, Basilar Impression, Retroflexed Odontoid, Brainstem Kinking and a disharmonic growth conflict between the neural axis and spine, with the retention of an apparently normal filum terminale on imaging.

None of these diseases has ever been related with this pathogenic mechanism, with the exception of the doctoral thesis “Contribution to the etiology of idiopathic syringomyelia” [26] in 1992, where, given the statistical evidence of a low position of the conus medullaris in Idiopathic Syringomyelia patients, has been postulated the existence of an axial caudal force affecting all of the human nervous system.

In our publications of 1996 [27, 28], the apparently normal *Filum terminale*, i.e. not showing any abnormality on imaging, is considered the transmitter or causative agent of the traction of the spinal cord and entire central nervous system, affecting them both and their bony surroundings - skull and spine, thus being the common cause of Arnold-Chiari Syndrome Type I, Idiopathic Syringomyelia, Idiopathic Scoliosis, Platybasia, Basilar Impression, Retroflexed Odontoid and Brainstem Kinking. We call *“Neuro-Cranio-Vertebral Syndrome”* the presence of one or more of these diseases in a patient, and when it is impossible to identify a traumatic, tumoral, infectious, obvious malformative congenital or any other cause, we call it *“Filum Disease”*.

Roth, in 1981 and 1986 [19, 20], proposed the disharmonic growth between the spine and the spinal cord to explain scoliosis and the Arnold-Chiari Syndrome I without any neurovertebral malformation. We regard this disharmonic growth, together with the mechanical conflict exerted by the seemingly normal Filum terminale, as causative of various idiopathic anomalies, such as Idiopathic Syringomyelia, Idiopathic Scoliosis, Basilar Impression, Retroflexed Odontoid, Platybasia and Brainstem Kinking.

Caudal traction is presumed to be present in all human beings from the ninth week of intrauterine life and any idiopathic Deviation of the Vertebral Column can be an expression of this same caudal traction force. Testut and Latarjet Péré’s quote from 1900 [30] is noteworthy: “*In 100 adults, examined regarding this topic* [referring to the lateral inflexions of the spine], *he confirmed their existence 93 times and the spine was straight only 7 times. The existence of lateral curvatures can therefore be considered normal*.” This indicates a potential prevalence of 93% of a coronal plane DVC not always perceived and therewith the possible existence of the axial caudal force and hence the Filum Disease. As Deviation of the Vertebral Column is one of the multiple ways through which Filum Disease can manifest itself, it would not be too adventurous to believe that there are signs on imaging and symptoms and signs in the clinical picture, in the majority of humans, usually unnoticed, that could confirm the possible universal existence of a mechanical conflict between the neuroaxis and the spine.

We are aware that patient selection depends on a previous diagnosis of certain conditions, initiated by health care professionals independent of our center, as well as on the patients’ assimilation of these diagnoses and of their proposed surgical solution or lack of therapeutic proposal. Although several factors can interfere in producing a selection bias (intensity of the clinical picture, individual temperamental and character features, regional cultural habits, ease of Internet use, social status, etc.) we can assume that even this search for a second opinion, as well as their understanding of the obvious limits of the current treatment, define the patients affected by this condition and therefore should not discourage any researcher trying to explain this clinical and imaging picture. Our patient sample’s representability for all population suffering with these conditions is not worrisome if one considers that it is merely the first description of a new pathology, awaiting future studies in which this global population should be better characterized in more objective conditions.

### Based on the results in 373 cases

#### Epidemiology

There is a predominance of the female gender (72%), the most frequent age to be diagnosed is 33 years (average 33.66 years, with standard deviation 15.87 years) and the time interval lapsed from onset until diagnosis is most often longer than 10 years, in 48% of cases.

#### Symptoms

The following principal symptoms define the clinical picture of FD in decreasing order of frequency: headache 84%, lumbosacral pain 72%, cervical pain 72%, balance alteration 72%, paresthesias 70%, thoracic back pain 65%, visual alterations 57%, lower extremity pain 56%, nervousness 53%, sphincter alterations 52%, fatigue 49%, upper extremity weakness 49%, nausea and/or vomiting 49%.

#### Signs

The most frequent signs in Filum Disease, in decreasing order of frequency, are: altered deep tendon reflexes in upper extremities 86%, altered deep tendon reflexes in lower extremities 82%, altered plantar reflexes 73%, decreased grip strength 70%, altered sensibility to temperature 69%, altered abdominal reflexes 68%, positive Mingazzini’s test 66%, altered sensibility to touch 65%, deviation of the uvula and/or tongue 64%, spontaneous nystagmus 55%, positive Romberg’s test 50%, positive straight leg-raising test 44%.

Significantly, the statistical analysis confirms the presence of correlations between Conus Medullaris level and Descent of cerebellar tonsils (*p* = 0.008) and between Conus Medullaris level and Deviation of the vertebral column (*p* = 0.045). It also confirms the existence of a correlation between Descent of cerebellar tonsils and Syringomyelia with cervical component (*p* = 0.003), coexisting with an interestingly inverse - that is, negative - association between Descent of cerebellar and “low” Syringomyelia (without cervical component, i.e. thoracic or thoracolumbar) (*p* = 0.001)*.* We also detected a positive correlation between Descent of cerebellar tonsils and Deviation of the vertebral column (*p* = 0.014).

Out of all symptoms and clinical signs, only the unilaterally or bilaterally positive straight leg-raising test (*p* = 0.048) and the unilaterally or bilaterally decreased grip strength (Kendall *p* = 0.019) have correlations with the level of conus medullaris. Altered deep tendon reflexes have correlations with Syringomyelia with cervical component (*p* = 0.005) and with Deviation of the vertebral column (*p* = 0.000).

#### Imaging

Relevant imaging features in decreasing order of frequency are: altered position of cerebellar tonsils 93% (Descent of cerebellar tonsils 73% and impaction Cerebellar Tonsils 20%); Low Conus Medullaris below T12-L1 88%; Deviation of the vertebral column in 76%; multiple disc disease 72%; syringomyelic cavity 52%.

## Conclusion

These observations are compatible with the conclusions of the thesis: the caudal traction force applied to the nervous system by means of the filum terminale is expressed in the form of Descent of cerebellar tonsils as the entire encephalon, including its lowermost part, the cerebellar tonsils, shift through the foramen magnum; the spine, seeking to minimize trauma on the spinal cord, bends and creates abnormal spinal curvatures such as scoliosis, kyphosis, hyperlordosis, rotoscoliosis, straightening; the central spinal cord tissue suffers ischemia and necrosis with production of a cavity filled with interstitial fluid or serum, the syringomyelic cavity; by acting on the skull and brainstem at the beginning of bone maturation, Platybasia, Basilar Impression, Retroflexed Odontoid and Brainstem Kinking develop. The course of the syringomyelic cavity is toward fistulization and mixing of the intracavitary fluid with cerebrospinal fluid, towards redilatation when there is a valvular mechanism or toward collapse and spinal cord atrophy.

Concluding a doctoral thesis and resulting published papers [26–28, 31, 32], we have proceeded thus to the description of a new nosological and etiopathogenic concept, proposing the pathological conditions of *Filum Disease*, when its origin is congenital, and *Neuro-Cranio-Vertebral Syndrome*, when the nature of the mechanical conflict between neuraxis and spine is acquired.

It is important for future studies to help understand better the origin of the axial caudal force, in order to assess the impact of the Filum Disease in the individual and in the population in general, which will help to comprehend the magnitude, consequences and anomalies of the asynchronic growth between neuraxis and spine on the central nervous system, skull and spine, as well as the rest of the organism. This knowledge would allow a new line of surgical and genetic treatments of these diseases. In the field of biology, it may be of interest to determine the presence of Filum Disease in other species belonging to the mammalia class, and also its absence in non-mammalian vertebrates.

The most relevant practical corollary of this paradigm shift is the solution that we propose in order to correct or prevent these abnormalities as soon as possible, consisting of the surgical intervention of *Filum terminale* sectioning. This is a minimally invasive procedure aimed at the release of this fibrous structure right above its coccygeal insertion, as it has been performed in most of the patients included in this report, as an alternative to many higher-risk and challenging surgical techniques tailored to solve these conditions [31, 32]. Of course, as they stand well out of the diagnostic and nosological purpose of this paper, these therapeutic innovations will be the subject of a future publication.

## Data Availability

The datasets used and/or analysed during the current study are available from the corresponding author on reasonable request.

## Abbreviations

ACSI: Arnold-Chiari Syndrome Type I
BI: Basilar Impression
BSK: Brainstem Kinking
CCD: Central Canal Dilatation
DCT: Descent of Cerebellar Tonsils
DVC: Deviation of the Vertebral Column
FD: Filum Disease
FM: Foramen magnum
FS®: Filum System®
IMC: Intramedullary Cyst
IS: Idiopathic Scoliosis
ISM: Idiopathic Syringomyelia
MRI: Magnetic resonance imaging
NCVS: Neuro-cranio-vertebral syndrome
LCM: Low-lying conus medullaris
PTB: Platybasia
R&D: Research & Development
RO: Retroflexed Odontoid
SCIE: Spinal Cord Ischemia-Edema
